# SLC38A6 expression in macrophages exacerbates pulmonary inflammation

**DOI:** 10.1186/s12931-023-02330-8

**Published:** 2023-01-27

**Authors:** Yizhao Peng, Weichao Chen, Fumeng Huang, Manman Geng, Xiaowei Li, Fujun Zhang, Wenhua Zhu, Liesu Meng, Rikard Holmdahl, Jing Xu, Shemin Lu

**Affiliations:** 1 Key Laboratory of Environment and Genes Related to Diseases, Xi’an Jiaotong University, Ministry of Education, Xi’an, Shaanxi 710061 China; 2 First Department of Respiratory Diseases, Xi’an Children’s Hospital, The Affiliated Children’s Hospital of Xi’an Jiaotong University, Xi’an, Shaanxi 710003 China; 3 Institute of Molecular and Translational Medicine (IMTM), and Department of Biochemistry and Molecular Biology, School of Basic Medical Sciences, Xi’an Jiaotong University Health Science Center, Xi’an, Shaanxi 710061 China; 4 National Joint Engineering Research Center of Biodiagnostics and Biotherapy, The Second Affiliated Hospital of Xi’an Jiaotong University, Xi’an, Shaanxi 710004 China; 5 Section for Medical Inflammation Research, Department of Medical Biochemistry and Biophysics, Karolinska Institute, 171 77 Stockholm, Sweden

**Keywords:** Pulmonary inflammation, Macrophage activation, SLC38A6, IL-1β

## Abstract

Pulmonary inflammation involves complex changes of the immune cells, in which macrophages play important roles and their function might be influenced by metabolism. Slc38a6 acts as a carrier of nutrient for macrophages (Mφ) to exert the function. In this study, pneumonia patient blood was found up-regulated SLC38A6 expression, which correlated with monocytes number and white blood cell number. The similar result was also shown in LPS induced sepsis mice. To reveal the key role of Slc38a6, we used systemic and conditional knock-out mice. Either systemic or Lyz^CRE^ specific knock-out could alleviate the severity of sepsis mice, reduce the proinflammatory cytokine TNF-α and IL-1β expression in serum and decrease the monocytes number in bronchial alveolar lavage and peritoneal lavage via flow cytometry. In order to reveal the signal of up-regulated Slc38a6, the Tlr4 signal inhibitor TAK242 and TLR4 knock-out mice were used. By blocking Tlr4 signal in macrophages via TAK242, the expression of Slc38a6 was down-regulated synchronously, and the same results were also found in Tlr4 knock-out macrophages. However, in the overexpressed Slc38a6 macrophages, blocking Tlr4 signal via TAK242, 20% of the mRNA expression of IL-1β still could be expressed, indicating that up-regulated Slc38a6 participates in IL-1β expression process. Collectively, it is the first time showed that an amino acid transporter SLC38A6 up-regulated in monocytes/macrophages promotes activation in pulmonary inflammation. SLC38A6 might be a promising target molecule for pulmonary inflammation treatment.

**Supplementary Information:**

The online version contains supplementary material available at 10.1186/s12931-023-02330-8.

## Introduction

Pneumonia is a common pulmonary infection that affects the alveoli and distal airways, it is a major health problem associated with high incidence rate and mortality in all age groups worldwide [1], and has been reported as the most common cause of death in children [2, 3]. The pathogenesis of pneumonia is a very complex process, involving the invasion of microorganisms into the lower respiratory tract, local skin infection and intestinal symbiotic bacteria infiltrating into the blood. Pulmonary inflammation is usually caused by two major factors, one is infection by microorganisms in the lung, the other is system infection associated with lung tissue [4]. Most hospital-acquired pneumonia (HAP) is caused by gram-negative bacteria (GNB) among bacterial pneumonia [2]. In the Chinese community-acquired pneumonia (CAP) statistical data of sputum culture from 2016 to 2017, GNB accounted for about 72.6%, suggesting that lipopolysaccharide (LPS), as a major pathogenic factor of GNB, is worthy of further study [5]. Bacterial pneumonia can be spread through inhalation or blood flow, the potent innate immune response generation in the lung during the non-pulmonary sepsis-associated pulmonary inflammation plays a crucial role in the disease outcome [6]. A clinical epidemiological study has found that the risk of pneumonia in critically ill patients recovering from the first severe sepsis (e.g., peritonitis) reaches about 40% [7], as a response to injury or pathogens, acute inflammation protects the host from systemic infection and restores tissue homeostasis. However, when the inflammation is not limited in magnitude or duration, it will lead to disease [8]. Therefore, there is an urgent need for a new point of view to better understand and effective treatment strategies to reduce pulmonary inflammation.

Pulmonary inflammation is related to numerous target and effector cells, which are mainly composed of immune cells such as macrophages, neutrophils, epithelial cells and endothelial cells [9]. The current research shows that due to the injury of perivascular epithelioid cells, these neutrophils and monocytes enter the alveoli through the lung epithelium, which induce pulmonary inflammation [10]. Pulmonary macrophages have been proposed to be the center to mediating and regulating inflammation. Recently, dramatic switches in cell metabolism accompanying these phenotypic and functional changes of macrophages also have been reported [11]. Although macrophages participate in pulmonary inflammation, it is not known how amino acid transporters influence their ability to respond to inflammation.

Solute carrier, also known as SLC, is responsible for the transmembrane absorption and flow of several substances, including amino acids, nucleotides, sugars, inorganic ions and drugs, as well as the excretion of toxic substances. The solute carrier family 38 (SLC38A, SNAT) comprises 11 members of the human genome and closely related mammalian genomes [12]. A functional characterization of SLC38A molecules is that they can balance various amino acids in the intracellular and extracellular base acid pool. Recent studies suggest that all SLC38A transporters are Na^+^ dependent and able to carry out neutral amino acids [13]. SLC38A2 senses and transports glutamine, regulates protein synthesis, cellular proliferation and apoptosis through the mechanistic (mammalian) target of rapamycin (mTOR) and general control nonderepressible 2 (GCN2) pathways [14]. SLC38A3 plays a role in developmental and epileptic encephalopathy by maintaining glutamine homeostasis [15]. SLC38A5 regulates mouse pancreas induced by glucagon receptor α cell function [16]. SLC38A6 is required in glutamine uptake and regulates the glutamine–glutamate cycle [17]. SLC38A7 is the primary lysosomal glutamine transporter required for the extracellular protein-dependent growth of cancer cells [18]. SLC38A9 transported arginine is required to efflux essential amino acids from lysosomes as mTORC1 activator [19]. SLC38A10 plays a role in the neurotransmission of neurons and astrocytes by transporting glutamine, glutamate and aspartate [20]. Although SNAT molecules play important roles in different cells, little was known of SLC38A6 in pulmonary inflammation.

Here, we demonstrate that up-regulated SLC38A6 plays an important role in sepsis-associated pulmonary inflammation through monocytes/macrophages. Our results show that not only the systematic Slc38a6 knock-out but also Slc38a6 specific knock-out in Lyz^CRE^ cells could ameliorate LPS induced sepsis-associated pulmonary inflammation severity, and decrease inflammatory cytokines, such as TNF-α and IL-1β. And up-regulated Slc38a6 is dependent Tlr4 signaling and critical for IL-1β expression. Taken together, our data firstly showed that an amino acid transporter Slc38a6 is involved in pulmonary inflammation via monocytes/macrophages activation dependent on Tlr4 signaling and could be a potential target for future development of therapeutic targets for pulmonary inflammation.

## Materials and methods

### Human materials

Pneumonia is defined in accordance with clinical criteria (Chest imaging examination showed patchy lung shadow or consolidation, pleural effusion, significant clinical body signs, the clinical diagnosis was bacterial infectious pneumonia and bronchopneumonia. Control group patients with hernia or slight bodily injury and patients who met the inclusion criteria were screened for eligibility within the first 24 h after they were admitted to the respiratory department of Xi’an Children's Hospital between September 2020 and April 2021. Blood samples were drawn from peripheral veins for each patient. The blood routine test was completed by the automatic blood cell analyzer (XS-500i, SYSMEX, Japan), blood routine examination can usually be divided into three categories: red blood cell (RBC), leukocyte, and platelet. CRP level test was completed by immunofluorescence analyzer (i-CHROMA Reader, Boditech MED Inc., Korea), procalcitonin level test was measured by fluorescence immune-quantitative analyzer (Getein1600, Getein Biotech Inc., China). Data including basic information, clinical features and laboratory indexes were collected from patients’ medical records. Demographic and clinical features include gender, age, chronic disease history, and so on. Information was extracted from the hospital database. A total of 67 patients were enrolled. Exclusion criteria were viral pneumonia (generally excluded according to symptoms, signs, season of onset, age and laboratory examination). Patients with underlying diseases, such as long-term use of immunosuppressants, tumors and congenital immune deficiency, were excluded. The basic characteristics of all patients are summarized in Additional file 1: Table S1. All participants gave their written informed consent prior to inclusion in the study. The study was approved by the Medical Ethics Committee of Xi’an Jiaotong University (No. 2021-223).

### Animals

All the mice were housed in a specific pathogen free facility with a 12-h light and dark at 22 ℃, and were supplied with clean food and water. Both male and female mice were used for the study. All animal experiments were approved by the Animal Advisory Committee at Xi’an Jiaotong University.

The Slc38a6 systemic knock-out mice and conditional knock-out mice were generated via CRISPR/Cas9 methods of deletion or editing on the 2nd exon of Slc38a6 gene by Shanghai Biomodel Organism Science & Technology Development Co., Ltd. Briefly, the F0 heterozygous were mice of Slc38a6^+/−^ and Slc38a6^fl/−^ were purchased and identified by PCR and sequencing. These founders were backcrossed with C57BL/6N.Q. mice or C57BL/6J mice for at least 6 generations. The Tlr4 knock-out mice and Lyz^CRE^ mice were purchased from Jackson laboratory which were housed in a specific pathogen free facility with a 12-h light and dark at 22 ℃, and were supplied with clean food and water [21]. Genotyping of these mice were routinely performed by DNA extraction of ear punched tissue, the representative graphs of DNA agarose gel electrophoresis and the specific primer sets used are shown in Additional file 1: Fig. S1 and Tables S2, S3.

### Animal model

C57BL/6J mice were purchased from Xi’an Jiaotong University animal center and were randomized to each group (n = 4). PBS or LPS (10 mg/kg, 20 mg/kg, *Escherichia coli* O111:B4, L2630, Sigma Aldrich) was given intraperitoneally to mice. These mice were used to induce sepsis-associated pulmonary inflammation model, and the expression level of Slc38a6 was measured.

Slc38a6^−/−^ and its littermate control mice were given intraperitoneal injection with 20 mg/kg LPS. These mice were used to study the effect of SLC38A6 deficiency on disease.

Slc38a6^fl/fl^Lyz^CRE^ and Slc38a6^fl/fl^ mice were given intraperitoneal injection with 30 mg/kg LPS. These mice were used to study the relationship between SLC38A6 and macrophages.

Model mice were intraperitoneally injected with 100 μL LPS solution comes from *Escherichia coli* O111:B4 (L2630, Sigma Aldrich) or PBS. For C57BL/6J mice model, 8–10 weeks old mice, half male and half female were selected, with LPS injection concentrations of 10 mg/kg and 20 mg/kg. For Slc38a6 knock-out mice, 8–10 weeks old mice, half male and half female were selected, with LPS injection concentration of 20 mg/kg. For Slc38a6 conditional knock-out mice, 8–10 weeks old mice, half male and half female were selected, with LPS injection concentration of 30 mg/kg. Mice were randomly assigned to treatment groups and all observations were performed by a double-blinded experimenter (blind to treatment and genotype). All control mice were from littermate mice. Mice were observed every 6 h for up to 72 h, the clinical score evaluation system referred to the method of Schappe [22]. The mice blood was taken after mice were euthanized. The peritoneal lavage fluid, bronchial lavage fluid, spleen and lung were obtained after mice were sacrificed.

### Cells

Bone marrow cells, isolated from Slc38a6-deficient mice and littermate heterozygous mice were seeded at the density of 2 × 10^6^/mL in RPMI 1640 containing 10% FBS and 20 ng/mL M-CSF for differentiating bone marrow–derived Mφ (BMDM).

Tlr4-deficient mice and littermate wide type mice were intraperitoneal injected with 3.8% thioglycolate solution to mice, PLF was taken after 24 h, then cells were seeded at the density of 2 × 10^6^/mL in RPMI 1640 containing 10% FBS after centrifuging, 24 h later, the suspended cells were removed to obtain peritoneal macrophages, and then stimulated with 100 ng/mL LPS for 24 h.

RAW264.7 cells were cultured in DMEM containing 10% FBS.

To overexpress human SLC38A6-v2 in RAW264.7 cell, the pLJM-hSLC38A6-v2, pMD2.G and psPAX2 plasmids were extracted by E.Z.N.A.™ Endo-free Plasmid Kit. And the packaged lentivirus in 293 T cell followed the described procedure [23]. The lentivirus medium was collected through 0.45 μm filter and then added to RAW264.7 cells. The positive cells were selected with 3 μg/mL puromycin for 7 days. And then the efficiency of overexpressed hSlc38a6 was determined by anti-FLAG via Western blotting.

All these cells were incubated at 37 °C in a humidified chamber supplemented with 5% CO_2_.

### Plasmid

The human Slc38a6 variant 2 CDS was cloned by PCR from human SLC38A6 cDNA ORF Clone vector (HG27257-U, Sino Biological, Inc., China) with hSlc38a6-v2 primer set. And the PCR product were purified and digested with NheI and XhoI, then ligased to pCDNA-3Flag vector via T4 ligase, the positive clones were sequenced by Sangon Biotech (Shanghai) Co., Ltd. Then the correct plasmid was digested with NheI and EcoRI, then ligased to pLJM-EGFP vector via T4 ligase, the positive clones were sequenced by Sangon Biotech (Shanghai) Co., Ltd.

### HE&IHC

Lungs were removed after the BAL fluid collection. The left upper lungs were fixed in 4% formaldehyde for 18–24 h and then embedded in paraffin. The paraffin-embedded lung sections (5 μm thickness) were cut and stained with hematoxylin and eosin. At least five visual fields with 100–200 μm of internal diameter were randomly collected and evaluated through a light microscope (× 200 magnification, Nikon, Tokyo, Japan). The inflammation score was based on a five-point scoring system: normal = 0; a few cells = 1; one layer of cell ring = 2; two to three layers of cell ring = 3; three to five layers of cell ring = 4; > 5 layers of cell ring = 5. Pulmonary interstitial inflammatory cell infiltration: take an area of 10 cm^2^ under 20 times of visual field, take a visual field for each sample, and randomly take 5 square areas under each visual field, and use Image J software to count the number of cells. The number of cells per unit area indirectly reflects the degree of interstitial inflammatory infiltration. Lung tissue sections were dewaxed and rehydrated in xylene and ethanol solutions. Antigen retrieval of these sections was performed by high pressure and endogenous peroxidase was blocked by 3% hydrogen peroxide. After being blocked by 5% bovine serum albumin for 30 min, the lung sections were incubated with primary antibodies (Abcam, ab237767) against Slc38a6 at 4 °C overnight. All sections were labeled by goat anti-rabbit and visualized using a DAB kit.

### Gene expression analysis

Total RNA was isolated either from tissue samples or cultured cells using TRI Reagent Solution. The RNA concentration was determined by NanoDrop (Thermo Fisher, USA). All the cDNA was synthesized by RevertAid First Strand cDNA Synthesis Kit (Thermo Fisher, USA) according to the manufacturer’s instructions. Quantitative RT-PCR (RT-qPCR) was performed by using Agilent optical system software (Agilent Technologies, Inc.) with Fast Start Universal SYBR Green Master (ROX) (Roche Diagnostics GmbH, Germany) for the relative mRNA expression of interested. The information of all the primers, products, and annealing temperatures is depicted in Additional file 1: Tables S2, S3. The 2^−ΔΔCt^ method was used to calculate relative gene expression levels. The mRNA expression levels of the target genes were normalized to *hprt* expression.

### ELISA

Detection of IL-1β and TNF-α in mice serum by ELISA (IL-1β ELISA kit, BMS6002, eBioscience; TNF-α ELISA kit, SMTA00B, R&D). Dilute the corresponding capture antibody in the coating buffer at 1:250, add 96-hole plates at 100 µL per hole, seal it with paraffin sealing film, and then incubate it at 4 ℃ overnight. The next day, wash the enzyme label plate with wash buffer, pipet 200 µL wash buffer into each hole, shake the plate and pour out the buffer, wash the plate three times. Then pipet 300 µL ELISA/ELISPOT Diluent to all wells and incubate the plate at RT for 1 h. Then wash the plate. Make a standard curve of 8 points. Dilute the mice serum 50–100 times by sample diluent, add 100 µL diluted serum sample into each well, pipet 50 µL biotinylated detection antibody to all wells and incubate the plate at RT for 2 h. Wash the plate. Add 100 µL prepared streptavidin HRP into each well and incubate the plate at 37 ℃ for half 1 h. Wash the plate 3–5 times with wash buffer. Add 100 µL TMB substrate application solution to all wells and incubate at RT for 15 min, avoiding direct exposure to intense light. Stop the enzyme reaction by quickly pipetting 100 µL of Stop Solution into each well, and read the O.D. value at 450 nm.

### Flow cytometry

The peritoneal lavage fluid from mice was collected by injection of 5 mL cold PBS into the peritoneal cavity. Peritoneal cells were obtained by centrifuging the peritoneal lavage fluid at 300*g*, 4 ℃ for 10 min.

The bronchial lavage fluid from mice was collected by injection of 1.5 mL cold PBS into the bronchial cavity. The bronchial lavage cells were obtained by centrifuging the fluid at 300*g*, 4 ℃ for 10 min.

The blood was collected from mice by cheek blooding and collected in 1% heparin EP tubes. And then the blood was added with ACK buffer, all the WBC were collected by 300*g*, 4 °C for 10 min.

The splenocytes from mice were collected in PBS by 40 μm cell strainer. And then the red blood cells were lysis by adding ACK buffer for 10 min, and splenocytes were collected by 300*g*, 4 °C for 10 min.

All these cells were counted and subjected to staining for flow cytometry. Firstly, cells were stained on ice with yellow fluorescent reactive dye (Live/dead Fixable Yellow Dead Cell Stain kit, L34968, Thermo) for 15 min and then added the antibodies cocktail (Ly6C-BV711, 128037, Biolegend; Ly6G-Pacific Blue, 127611, Biolegend; F4/80-FITC, 123108, Biolegend; CD11b-APC, 101212, Biolegend; CD45.2-BV785, 109839, Biolegend) for staining another 30 min. Wash the cell with PBS for 3 times, and then BD Cytofix/Cytoperm was used to further stain iNOS (iNOS-PE-cy7, 25-5920-80, eBiolegend) for 20 min on ice. Stained cells were washed with PBS for 2 times and then washed again with 1 mL of cold FACS buffer, resuspended in 200 μL of FACS buffer, and placed on ice for immediate analysis by FACS (ACEA Biosciences Inc., USA). All data were analyzed by FlowJo version 10 software.

### Statistics

All data were presented as mean ± SEM. The calculation for statistical significance was measured using two-tailed Student’s t test or one-way ANOVA among groups. Survival rates were analyzed using the log-rank test. Logistic regression analysis was used to examine covariate-adjusted associations among groups. All statistical analyses were performed using Prism 8.0 software (GraphPad Software Inc.), and *p* values less than 0.05 were considered statistically significant.

## Results

### The expression of SLC38A6 is up-regulated in PBMCs of patients with bacterial infection

**Fig. 1:**
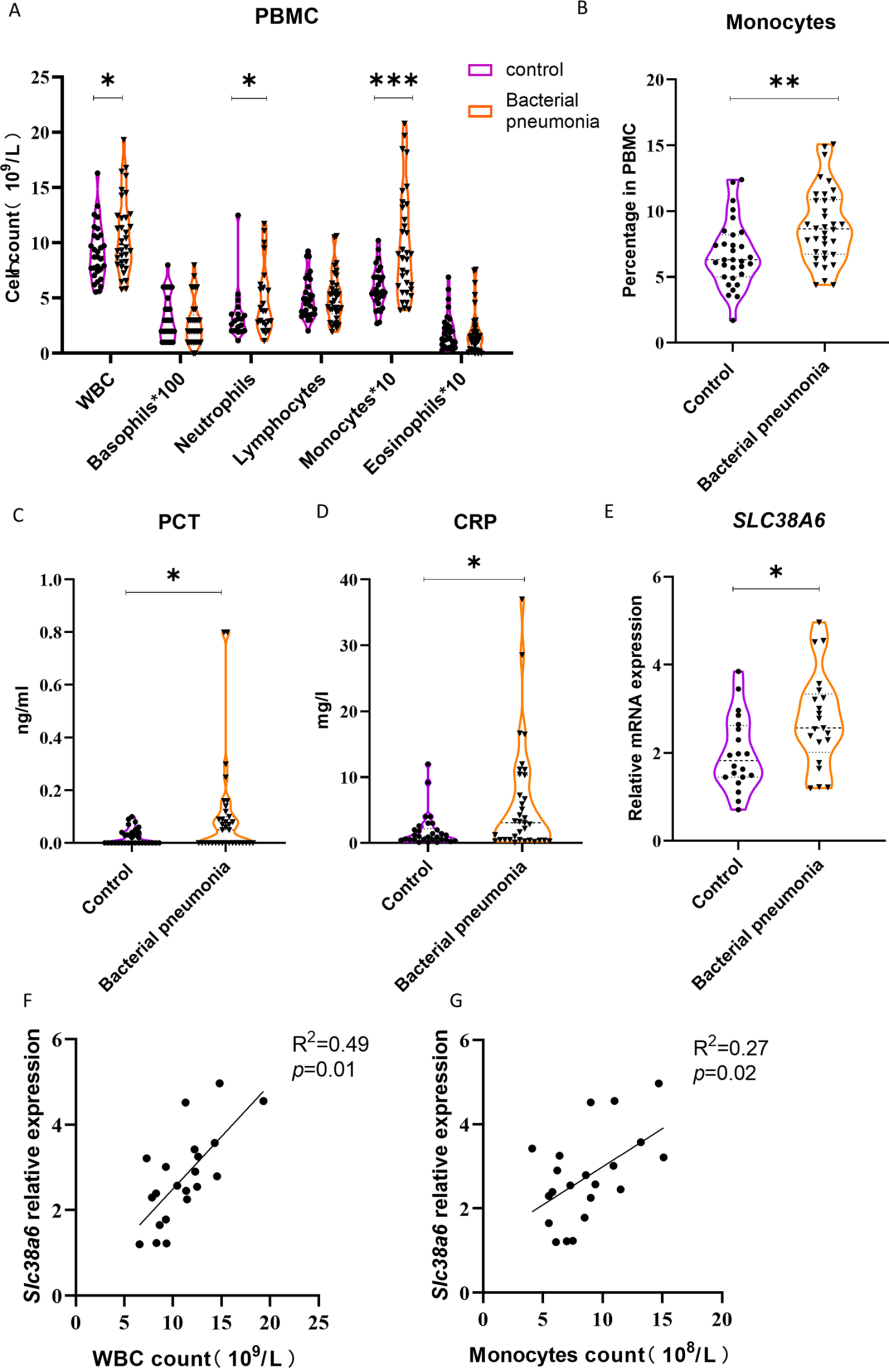
The expression of *SLC38A6* was up-regulated in bacterial pneumonia patients. PBMCs counts (**A**) between control patients (n = 31) and bacteria pneumonia patients (n = 36). Percentage of monocytes (**B**) between control patients (n = 31) and bacteria pneumonia patients (n = 36). PCT and CRP level (**C**) between control patients (n = 31) and bacteria pneumonia patients (n = 36). *SLC38A6* expression level in PBMC cells (**D**) between control patients (n = 20) and bacteria pneumonia patients (n = 21). **E** Correlation analysis of *SLC38A6* expression level with WBC and monocytes count (n = 21). Data are presented as mean ± SEM of independent experiments. *p < 0.05, **p < 0.01 and ***p < 0.001

Peripheral blood mononuclear cells (PBMCs) were collected from patients with bacterial pneumonia or control group patients (with hernia or slight bodily injury). Patients with bacterial pneumonia was significantly increased the number of leukocytes, neutrophils and monocytes than the control group (Fig. 1A), as well as the proportion of monocytes was increased significantly (Fig. 1B). Meanwhile, the concentrations of procalcitonin (PCT) and C-reactive protein (CRP) were increased significantly compared with the control group (Fig. 1C, D) In the meantime, the PBMCs mRNA level of *SLC38A6* expression was significantly up-regulated in patients with bacterial pneumonia compared with the control group (Fig. 1E). In addition, the *SLC38A6* expression level was positively correlated with the number of monocytes (R^2^ = 0.27, p = 0.02) and WBC (R^2^ = 0.49, p = 0.01) (Fig. 1F, G), suggesting that the overexpressed *SLC38A6* may participate in bacterial pneumonia.

### The expression level of *Slc38a6* was highly-expressed in sepsis-associated pulmonary inflammation mice model

**Fig. 2:**
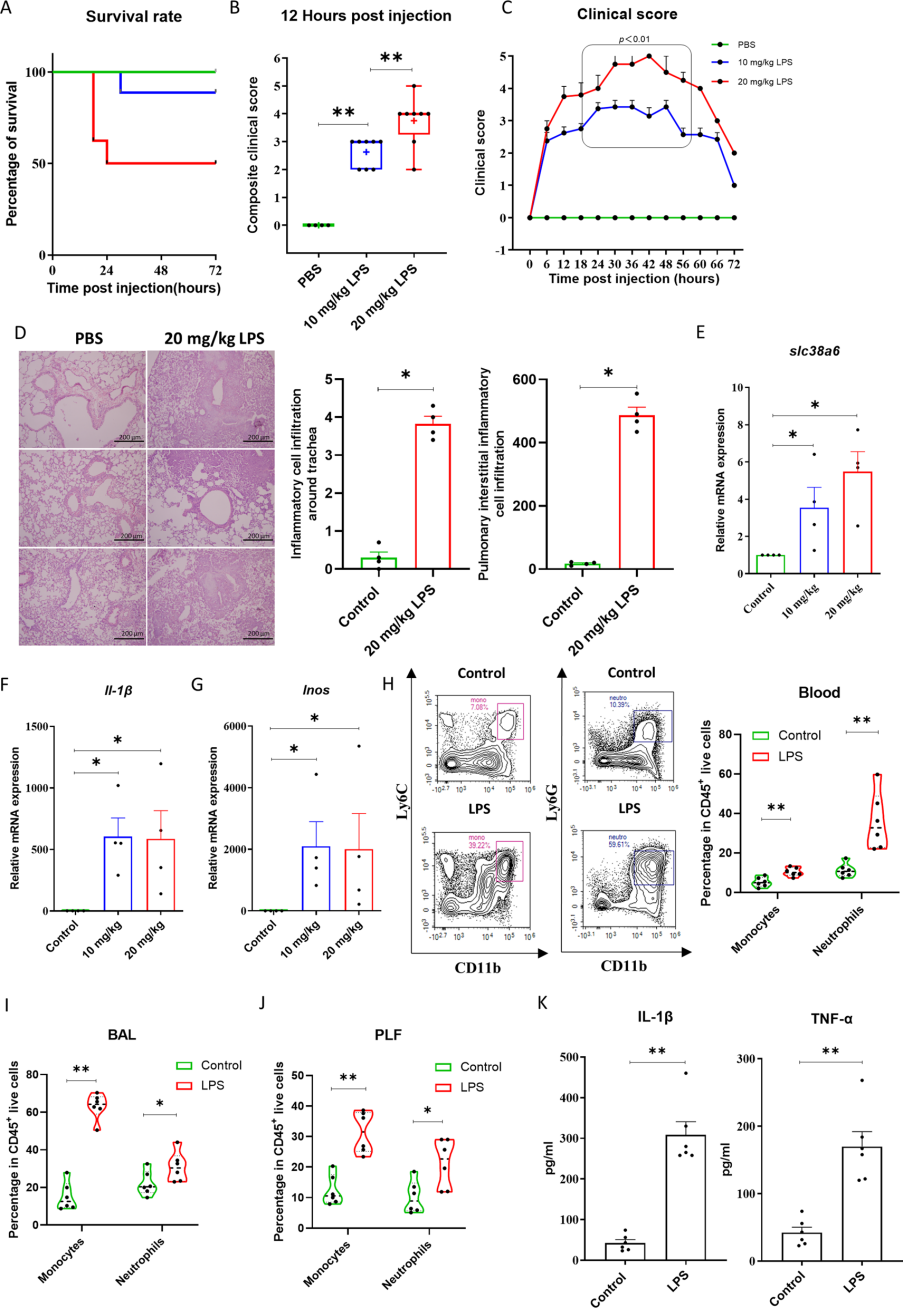
The expression level of *Slc38a6* was highly-expressed in sepsis-associated pulmonary inflammation mice. Survival rate (**A**) and clinical score (**B**, **C**) among control group (n = 4), 10 mg/kg LPS i.p. group (n = 8) and 20 mg/kg LPS i.p. groups (n = 8). **D** HE staining samples of lung tissue between control group and 20 mg/kg LPS i.p. group, the histological scores of inflammatory cell infiltration (n = 4). *Slc38a6* (**E**), *Il-1β* (**F**), *Inos* (**G**) relative expression level in PLF among three groups (n = 4). Neutrophil and monocyte percentage in blood (**H**), BALF (**I**), PLF (**J**) at 24 h post injection between PBS and 20 mg/kg LPS group by FACS (n = 6). **K** IL-1β and TNF-α concentration in serum at 24 h post injection between PBS and 20 mg/kg LPS group by ELISA (n = 6). Data are presented as mean ± SEM of independent experiments. *p < 0.05 and **p < 0.01

In order to mimic sepsis-associated pulmonary inflammation, mice were injected with PBS, 10 and 20 mg/kg LPS into peritoneal cavities to induce sepsis, respectively. All the mice were monitored every 6 h. The mortality and disease score of mice were increased significantly along with the LPS concentration (Fig. 2A–C). The pulmonary infiltration of inflammatory cells in the lung was obvious after LPS stimulation (Fig. 2D). We also found the cytokine storm molecular, *Il-1β*, *Inos* was increased significantly after LPS priming (Fig. 2F, G), and LPS stimulate concentration was synchronized with the up-regulation of *slc38a6* mRNA expression in total peritoneal lavage fluid cells (Fig. 2E). Based on the above LPS concentration results, we induced a 24h short term model to analyze cells, we obtained mice bronchoalveolar lavage fluid (BALF), peritoneal lavage fluid (PLF) and blood, found that the percentage and cell count of neutrophils and macrophages/monocytes were significantly upregulated after LPS stimulation (Fig. 2H, I, G). Meanwhile, serum IL-1β*,* and TNF-α were significantly up-regulated in PLF by ELISA (Fig. 2K), as well as IL-6 (Additional file 1: Fig. S2A).

### Slc38a6 deficiency protects mice from sepsis-associated pulmonary inflammation

**Fig. 3:**
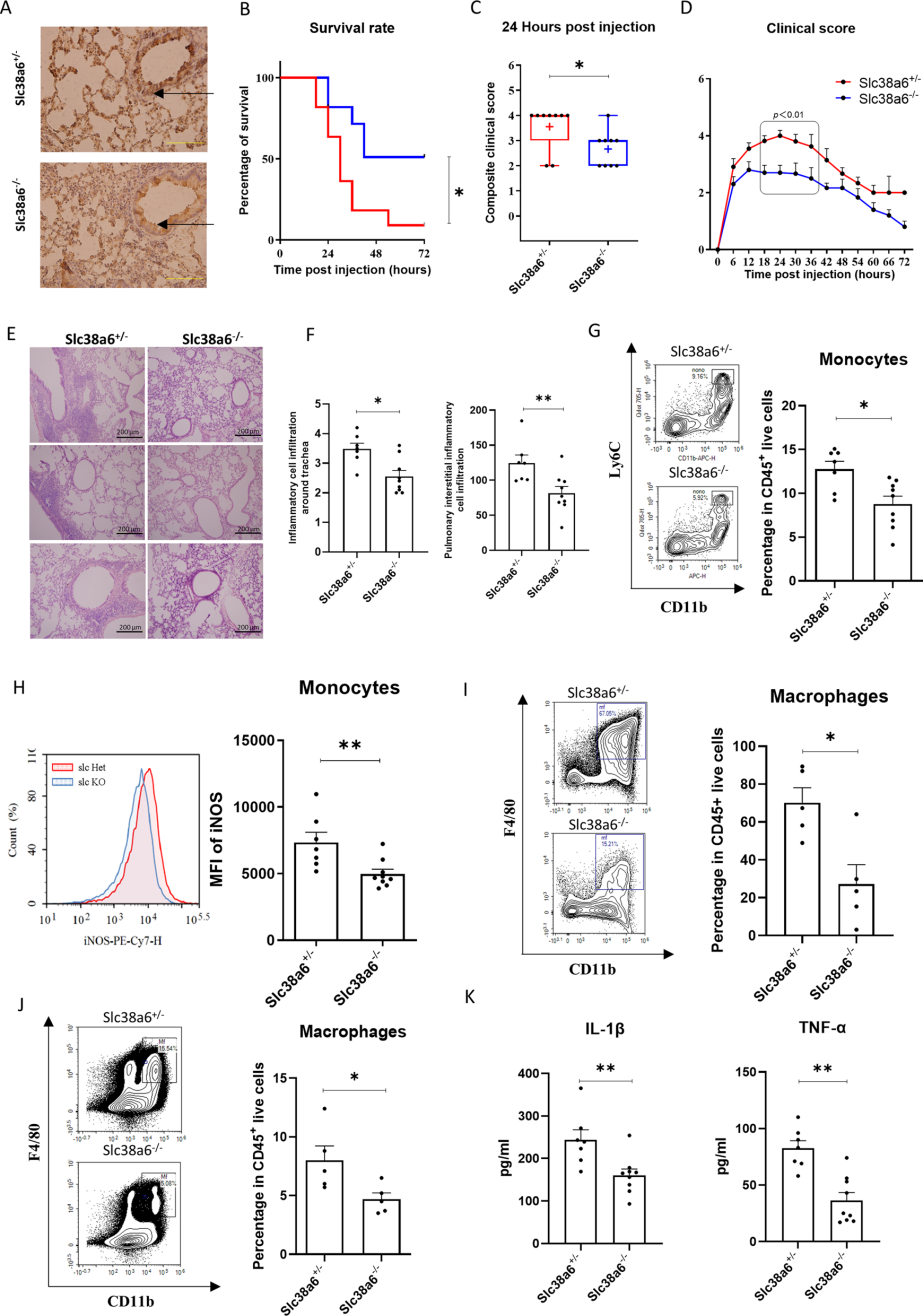
Slc38a6 deficiency protects mice from sepsis-associated pulmonary inflammation. **A** IHC for Slc38a6 in lung tissue between Slc38a6^+/−^ and Slc38a6^−/−^ mice. Survival rate **B** and clinical scores (**C**, **D**) between Slc38a6^+/−^ and Slc38a6^−/−^ group (n = 11). HE staining samples (**E**) of lung tissue between Slc38a6^+/−^ and Slc38a6^−/−^ group (n = 3) and the histological scores (**F**) of inflammatory cell infiltration (n = 7 or 9). Blood monocyte percentage (**G**) and MFI of blood monocyte iNOS at 24 h post injection (**H**) (n = 7 or 9). Macrophage percentage in BALF (I) and splenocytes (**J**) at 72 h post injection (n = 5). **K** IL-1β, TNF-α concentration in serum between Slc38a6^+/−^ and Slc38a6^−/−^ group at 24 h post injection (n = 7 or 9). Data are presented as mean ± SEM of independent experiments. *p < 0.05 and **p < 0.01

To further understand the up-regulated *Slc38a6* function in LPS-induced pulmonary inflammation, a *Slc38a6* knock-out mice was designed. Lacking of the second exon of *slc38a6* gene in mice, the *Slc38a6* expression could not be detected either in mRNA or protein level (Fig. 3A). Then the LPS-induced pulmonary inflammation was applied in Slc38a6 and its littermate Slc38a6^+/−^ mice. The results showed that Slc38A6^−/−^ mice had a higher survival rate, which increased 4 folds compared with littermate Slc38A6^+/−^ mice. The onset was also delayed when it in the Slc38A6 deficient mice (Fig. 3B). Meanwhile, Slc38A6^−/−^ mice showed a less severe disease score during the acute phase (Fig. 3C, D). The pulmonary inflammatory infiltration in the lung was also relieved in Slc38A6^−/−^ mice, compared with Slc38A6^+/–^ mice (Fig. 3E, F). In order to further clarify the relevant infiltrating immune cells, FACS was performed on mice. The results showed that the MFI of monocytes iNOS decreased significantly in the peripheral blood of Slc38A6^−/−^ mice, accompanied by the decrease of monocyte proportion (Fig. 3G, H). The same trend was found in BALF and spleen macrophages (Fig. 3I, J). Alongside the decrease of proportion, the total number of macrophages in BALF and spleen also decreased in Slc38A6^−/−^ mice (Additional file 1: Fig. S7A, 7B). In addition, IL-1β, TNF-α in serum also decreased significantly in Slc38A6^−/−^ mice, compared with Slc38A6^+/–^ mice (Fig. 3K).

### Slc38a6 plays the role of aggravating disease through macrophages

**Fig. 4:**
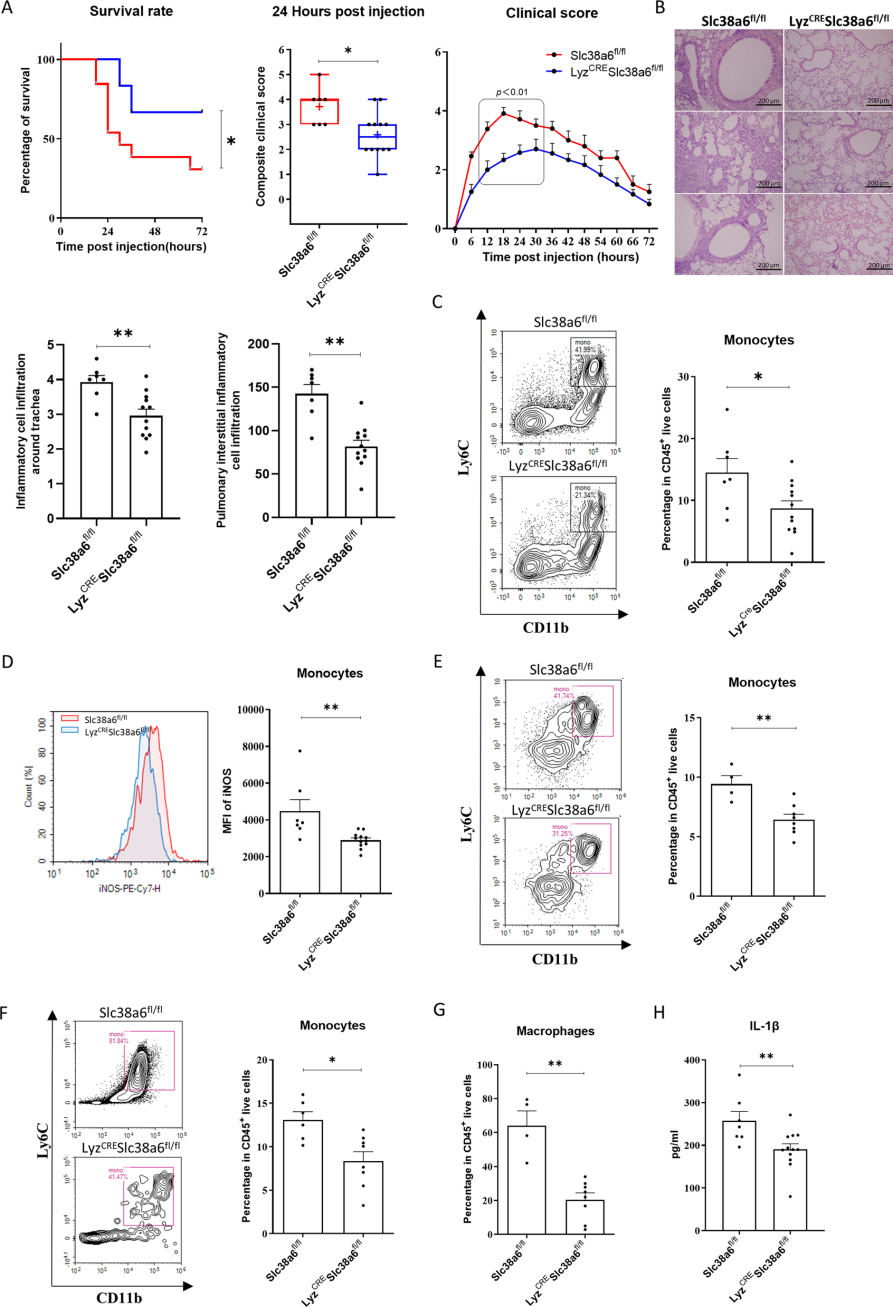
The exacerbating effect of Slc38a6 was mainly through macrophages. **A** Survival rate and clinical score between Lyz^CRE^Slc38a6^fl/fl^ and Slc38a6^fl/fl^ group (n = 13 or 12). **B** HE staining samples of lung tissue between between Lyz^CRE^Slc38a6^fl/fl^ and Slc38a6^fl/fl^ group and histological scores of inflammatory cell infiltration from 24 to 72 h after injection (n = 7 or 12). Blood monocyte percentage **C** and MFI of blood monocyte iNOS (**D**) at 24 h post injection (n = 7 or 12). Monocyte/macrophage percentage in PLF (**E**), in splenocytes (**F**) and in BALF (**G**) at 72 h post injection (n = 4–6 or 8) by FACS. **H** IL-1β concentration in serum between Lyz^CRE^Slc38a6^fl/fl^ and Slc38a6^fl/fl^ group at 24 h post injection by ELISA (n = 7 or 12). Data are presented as mean ± SEM of independent experiments. *p < 0.05 and **p < 0.01

Through systemic deletion of *Slc38a6*, mice could alleviate the sepsis-associated pulmonary inflammation, mainly by reducing cytokine storm and activated macrophages, monocytes or neutrophils. Further interested us was whether the deletion of *Slc38a6* only in monocytes and macrophages could help the disease? So a Lyz-CRE mouse were used for conditional knocked out the *Slc38a6* in Lyz positive expressed cell. After being induced by LPS, Lyz positive *Slc38a6* deletion mice showed a higher survival rate, which was increased by 2 folds compared with its littermates Slc38A6^fl/fl^ mice, and the onset of the disease was delayed for 12 h. Meanwhile, Lyz^CRE^Slc38A6^fl/fl^ mice showed a less severe disease symptom than Slc38A6^fl/fl^ mice (Fig. 4A). The pulmonary inflammatory infiltration was also relieved in Lyz^CRE^Slc38A6^fl/fl^ mice, compared with Slc38A6^fl/fl^ mice (Fig. 4B). Then the related immune cells were investigated by FACS. Compared with Slc38A6^fl/fl^ mice, the proportion of monocytes in peripheral blood of Lyz^CRE^Slc38A6^fl/fl^ mice was decreased significantly (Fig. 4C), the similar results were also found in PLF (Fig. 4E). In BALF, the proportion of macrophages found decreased significantly in Lyz^CRE^Slc38A6^fl/fl^ mice compared with Slc38A6^fl/fl^ mice (Fig. 4F), so as splenocytes (Fig. 4G). Meanwhile, the total number of macrophages in the spleen and BALF also decreased in Lyz^CRE^Slc38A6^fl/fl^ mice (Additional file 1: Fig. S7C, 7D). We found that the MFI of monocytes iNOS decreased significantly in the peripheral blood of Lyz^CRE^Slc38A6^fl/fl^ mice (Fig. 4C), serum IL-1β of Lyz^CRE^Slc38A6^fl/fl^ mice reduced (Fig. 4H). These results suggested loss of *Slc38a6* in Lyz positive cells could decrease the monocyte activation.

### Slc38a6 promotes inflammation by affecting macrophage function

**Fig. 5:**
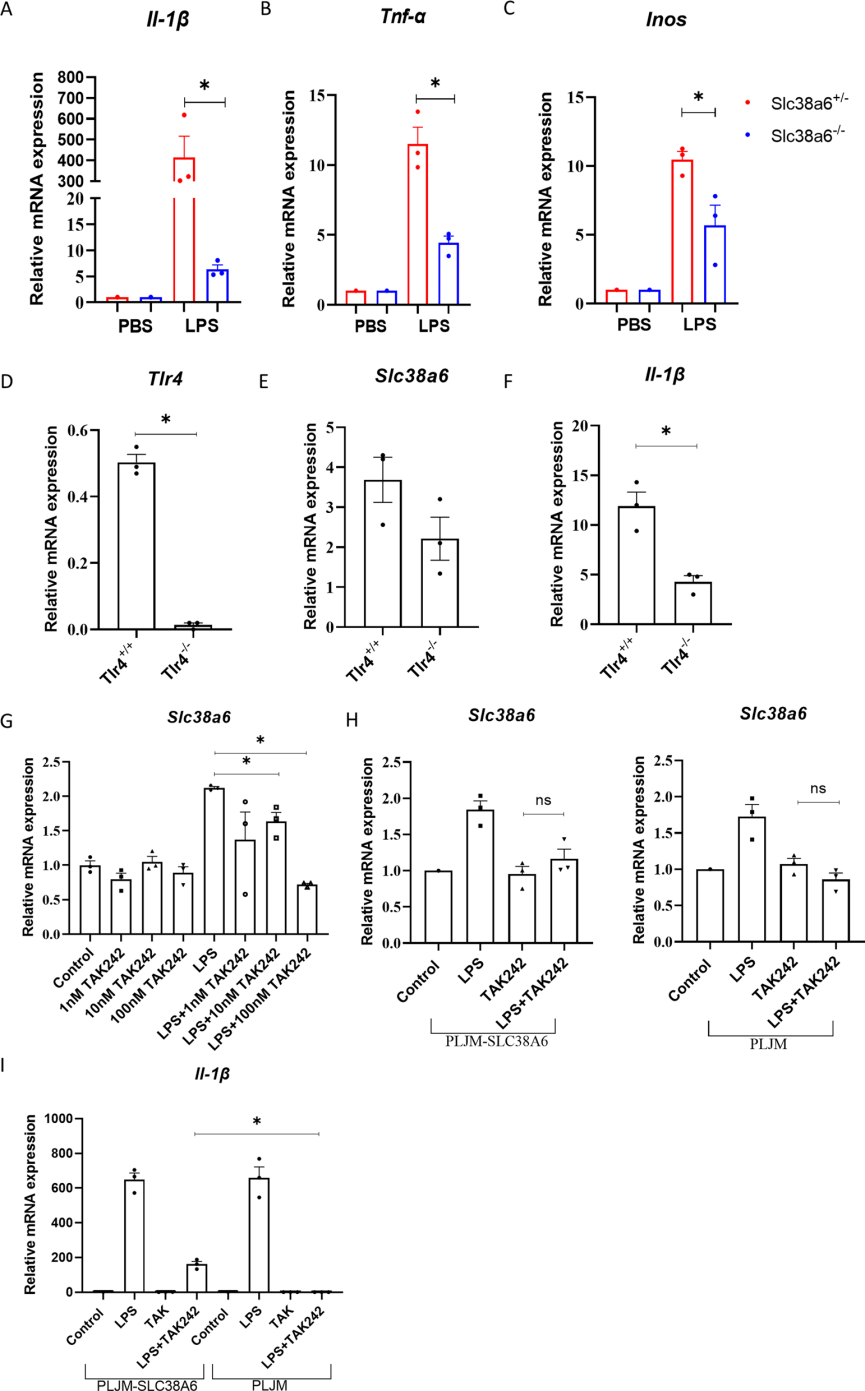
Slc38a6 promotes inflammation by affecting macrophage function. *Il-1β* (**A**), *Tnf-α* (**B**), *Inos* (**C**) relative expression level detected by RT-qPCR between SLC38A6^+/−^ and SLC38A6^−/−^ stimulated BMDM (n = 3). *Tlr4* (**D**), *Slc38a6* (**E**) and *Il-1β* (**F**) expression level between Tlr4^+/−^ and Tlr4^−/−^ stimulated peritoneal macrophages. **G** *Slc38a6* expression level by LPS stimulation with or without TAK242 in RAW264.7. *Slc38a6* (**H**) and *Il-1β* (**I**) relative expression level between PLJM-SLC38A6 and PLJM cells. Data are presented as mean ± SEM of three independent experiments. *p < 0.05

Since Lyz^CRE^ Slc38a6 knock-out mice reduced IL-1β expression, the next question was how up-regulated Slc38a6 was involved in monocyte/macrophage activation. The bone marrow-derived macrophages were cultured and stimulated with LPS. As the results shown in Fig. 5A–C, the mRNA level of *Inos*, *Il-1β* and *Tnf-α* was significantly decreased in Slc38a6 deficiency macrophages. By considering the sepsis was caused by LPS, LPS receptor TLR4 signaling was detected via Tlr4 signaling inhibitor TAK242 and Tlr4 knock-out macrophages. Firstly, different concentrations of TAK242 were used for revealing the up-regulated *Slc38a6* expression after LPS priming. As the results Fig. 5G and H showed after blocking the TLR4 via 10 nM or 100 nM of TAK242, the up-regulated mRNA level of *Slc38a6* was decreased significantly, the similar result also be found in *Tlr4* knock-out macrophages, suggesting that the activation of *Tlr4* signaling was responsible for up-regulating *Slc38a6* in macrophages (Fig. 5D–F). Interestingly, when overexpressed human Slc38a6 CDS in mice macrophages cell line RAW264.7, the overexpressed Slc38a6 was responsible for maintaining 20–25% of Il-1b expression (Fig. 5I). What’s more, the mRNA level of *Slc38a6*, *Il-1β* and *Tnf-α* were also detected by different time points after LPS stimulation. As results showed in Additional file 1: Fig. S2B, C, the mRNA level of *Slc38a6* expression trend was the same as *Il-1β*, different from the mRNA level of *Tnf-α* expression trend. These results suggested that Tlr4 respond to LPS stimulation and up-regulated Slc38a6 was involved in maintaining the proinflammatory cytokine *Il-1β* expression.

## Discussion

Through our study, we conclude that the expression of SLC38A6 is enhanced in PBMCs of patients with bacterial pneumonia and in the mice model of sepsis-associated pulmonary inflammation. Deficiency of SLC38A6 can reduce the severity and inflammation of sepsis-associated pulmonary inflammation in mice, which mainly depends on the participation of macrophages. SLC38A6 participates in the occurrence of inflammation by participating in the regulation of IL-1β in macrophages, which is in a TLR4 dependent manner. To our knowledge, our study is the first time to report the role of SLC38A6 in patients with pneumonia and sepsis-associated pulmonary inflammation in mice, which provides a new possibility for the study of the function of amino acid transporters, and also provides a potential molecular target for the treatment of bacterial pneumonia and sepsis-associated pulmonary inflammation.

To study pulmonary inflammation, we collected PBMC samples from patients with bacterial pneumonia and control patients, found increased Slc38a6 expression, and the upregulated expression level in PBMCs was positively correlated with the increasing number of monocytes. We know that pulmonary macrophages account for 90–95% of pulmonary immune cell homeostasis, they play an important role in the initiation and regression of pulmonary inflammation [24, 25]. Our results reveal that the expression of SLC38A6 is up-regulated in PBMCs of patients with bacterial pneumonia, which is the first report of this molecule in pneumonia, SLC38A6 family molecules are generally considered to be related to amino acid transport function, so we speculate that SLC38A6 may participate in pulmonary inflammation by affecting the synthesis or modification of inflammatory factors. Meanwhile, we found that the frequency change of monocytes was positively correlated with the gene expression of SLC38A6, and speculated that Slc38a6 might play a role through monocytes, although we could not directly detect the expression of Slc38a6 in human purified blood monocytes/tissue macrophages. For understanding the protentional Slc38a6 function, we focused our research on sepsis-associated pulmonary inflammation.

In the study of sepsis-associated pulmonary inflammation, commonly used animal models generally include LPS, CLP, and bacterial infection. As a single, simple and clear bacterial component, LPS is the main toxic substance of gram-negative bacteria. We used LPS induced sepsis-associated pulmonary inflammation model, which is more in line with our research plan and more conducive to the study of the function of SLC38A6 [26, 27]. At the early stage of animal model construction, due to the different strains of mice, LPS concentration is vary, so we tested 10, 20 and 30 mg/kg, finally we selected 20 mg/kg LPS intraperitoneal injection concentration on B6 background mice and 30 mg/kg LPS intraperitoneal injection concentration on B6N.Q. background mice. At this concentration, the symptoms of mice are obvious and the mortality is moderate. We stimulated SLC38A6^+/−^ and SLC38A6^−/−^ mice with LPS, the main pathogenic factor of GNB, to mimic sepsis-associated pulmonary inflammation [28–30]. Interestingly, when mice were deprived of SLC38A6, systemic symptoms and pulmonary inflammation were both alleviated. In addition, studies have shown that the reduction of peripheral blood monocyte may reduce pulmonary inflammation [31], this is similar to our results, we found that the proportion of monocytes/macrophages were significantly down-regulated, and macrophage activation markers iNOS was also significantly down-regulated, suggesting that when stimulation occurs, monocytes/macrophages may reduce classical activation (M1) due to the absence of SLC38A6, thus reducing the level of inflammation [32–34]. According to the analysis of experimental results, we believe that this may be related to the involvement of SLC38A6 in the regulation of macrophage function. Therefore, the research on sepsis-associated pulmonary inflammation is of great significance, and it is necessary to continue our research. In addition, because we did not directly detect the Slc38a6 expression level in the purified monocytes/macrophages, we only obtained relevant conclusions. We wondered whether the correlation between the expression of Slc38a6 and the significant increase of monocytes in peripheral blood could be used as a hint of pulmonary inflammation, perhaps Slc38a6 can participate in immunity by regulating the function of immune cells such as macrophage.

Based on the above results, we would like to study whether SLC38A6 affects the occurrence and development of inflammation through monocytes/macrophages. By inducing model in Lyz^CRE^SLC38A6^fl/fl^ mice and SLC38A6^fl/fl^ mice, the results showed that the survival rate of Lyz^CRE^SLC38A6^fl/fl^ mice was higher, the clinical symptoms of the mice were milder, and their pulmonary inflammatory infiltration was relieved, compared with SLC38A6^fl/fl^ mice. These results are consistent in Slc38a6 knock-out mice, which proves that SLC38A6 may participate in inflammation through Lyz^+^ cells. It revealed that SLC38A6 is mainly involved in the occurrence of inflammation by participating in the function of Lyz positive cells. Besides, we found that the MFI of iNOS in monocytes in peripheral blood of Lyz^CRE^SLC38A6^fl/fl^ mice decreased significantly, indicating that the deletion of SLC38A6^fl/fl^ in Lyz positive cells could reduce the activation of monocytes. Interestingly, we found that in Lyz^CRE^SLC38A6^fl/fl^ mice, IL-1β in serum significantly reduced, but not TNF-α. It is important to point out that we found neutrophils proportion upregulated after LPS priming in mice, and Lyz is also expressed in neutrophils, we cannot rule out that Slc38a6 also played a part of the auxiliary role through neutrophils. These results suggest that SLC38A6 plays an important role in monocytes/macrophages function, involving classical activation and cytokines, especially IL-1β. IL-1β is related to the characteristic pathological changes of the airway in patients with chronic obstructive pulmonary disease, chronic pulmonary inflammation and lung tissue damage [35]. These results provide us with the possibility that we can target SLC38A6 to treat sepsis-associated pulmonary inflammation by affecting macrophage function.

Next, we focused on the mechanism of up-regulated SLC38A6 on monocytes/macrophages. By using TAT242, an inhibitor of TLR4 signaling, the up-regulated Slc38a6 mRNA expression was blocked. The similar results also were found in TLR4 knock-out macrophages. These results indicated that the up-regulated Slc38a6 were due to TLR4 signaling. Interestingly, another transporter, SLC37A2 is also reported up-regulated in LPS stimulated macrophages, reduces LPS-induced pro-inflammatory cytokine expression via reduced glycolysis [36]. Slc38a6 belongs to the neutral amino acid transporters, which could transport glutamine into the cell. Glutamine plays an essential role in both glucose and amino acid metabolism. And it was also a resource of α-ketoglutarate. Key TCA metabolite α-ketoglutarate orchestrates macrophage activation via PHD-dependent post-translational regulation of IKK-β, affecting IL-1β and TNFα expression. [37]. IL-1β was suggested to be associated with both the innate and adaptive immune response, and a consequent increased susceptibility to infections [38]. It plays a central role in inflammation and has also been shown to drive epigenetic reprogramming of monocytes, and plays a key role in training immunity, especially in macrophages [39, 40]. Here, we also reported that in over-expressed Slc38a6 macrophages, blocking Tlr4 signaling only could reduce 75–80% of IL-1β expression, indicating that SLC38A6 is one of the controllers for macrophages to produce proinflammatory cytokines.

## Conclusion

We have found a new solute carrier called SLC38A6, which is essential for sepsis-associated pulmonary inflammation, SLC38A6 may mediate inflammation by directly participating in the maintenance of IL-1β in macrophages. It is speculated that SLC38A6 may affect kinase activity by changing the transport of amino acids type or concentration, which may be very important for the expression of inflammatory genes in macrophages. In addition, these findings indicate that SLC38A6 can become a pharmacologically manageable molecular target for developing new treatment strategies for bacterial pneumonia and sepsis-associated pulmonary inflammation.

## Supplementary Information

**Additional file 1: Table S1.** Clinical characteristics of patients. **Table S2.** The information of primers, products, and annealing temperatures. **Table S3.** The information of mice genotyping primers, products, and hSlc38a6-v2 primers. **Figure S1.** The agarose gel electrophoresis for genotyping. (A) Slc38a6 knock-out mice genotyping. (B) Slc38a6 conditional knock-out mice genotyping. (C) Lyz-CRE mice genotyping. (D) Tlr4 knock-out mice genotyping. **Figure S2.** (A) *Il-6* relative expression level in PLF among control group (n = 4), 10 mg/kg LPS i.p. group (n = 4) and 20 mg/kg LPS group (n = 4). (B) TNF-α concentration in serum between Lyz^CRE^Slc38a6^fl/fl^ and Slc38a6^fl/fl^ group at 24 h post injection by ELISA (n = 7 or 12). (C) Relative expression of *Il-1β* and *Slc38a6* after LPS stimulation in RAW264.7. (D) Relative expression of *Tnf-α* and *Slc38a6* after LPS stimulation in RAW264.7. Data are presented as mean ± SEM of independent experiments. *p < 0.05. **Figure S3.** (A) Flow cytometry strategy for peripheral blood. (B) Staining of unstained samples with different antibodies. **Figure S4.** (A) Flow cytometry strategy for spleen cells. (B) Staining of unstained samples with different antibodies. **Figure S5.** (A) Flow cytometry strategy for PLF. (B) Staining of unstained samples with different antibodies. **Figure S6.** (A) Flow cytometry strategy for BAL fluid. (B) Staining of unstained samples with different antibodies. **Figure S7.** Macrophage percentage in BALF between Slc38a6^+/−^ and Slc38a6^−/−^ mice (A) and splenocytes (B) at 72 h post injection (n = 5). Monocyte/macrophage percentage in splenocytes (C) and in BALF (D) at 72 h post injection (n = 4–6 or 8).

## Data Availability

The datasets used and/or analyzed during the current study are available from the corresponding author on reasonable request.

## Abbreviations

Mφ: Macrophage
HAP: Hospital-acquired pneumonia
GNB: Gram-negative bacteria
CAP: Community-acquired pneumonia
BMDM: Bone marrow-derived macrophage
PBMC: Peripheral blood mononuclear cell
PCT: Procalcitonin
CRP: C-reactive protein
BALF: Bronchoalveolar lavage fluid
PLF: Peritoneal lavage fluid
