# Supplementary material for: SLC38A6 expression in macrophages exacerbates pulmonary inflammation
Source: Respir Res. 2023 Jan 27;24:33. doi: 10.1186/s12931-023-02330-8 (PMC9881254; doi:10.1186/s12931-023-02330-8)

Table S1

Clinical characteristics of patients.

Table S2

The information of primers, products, and annealing temperatures.


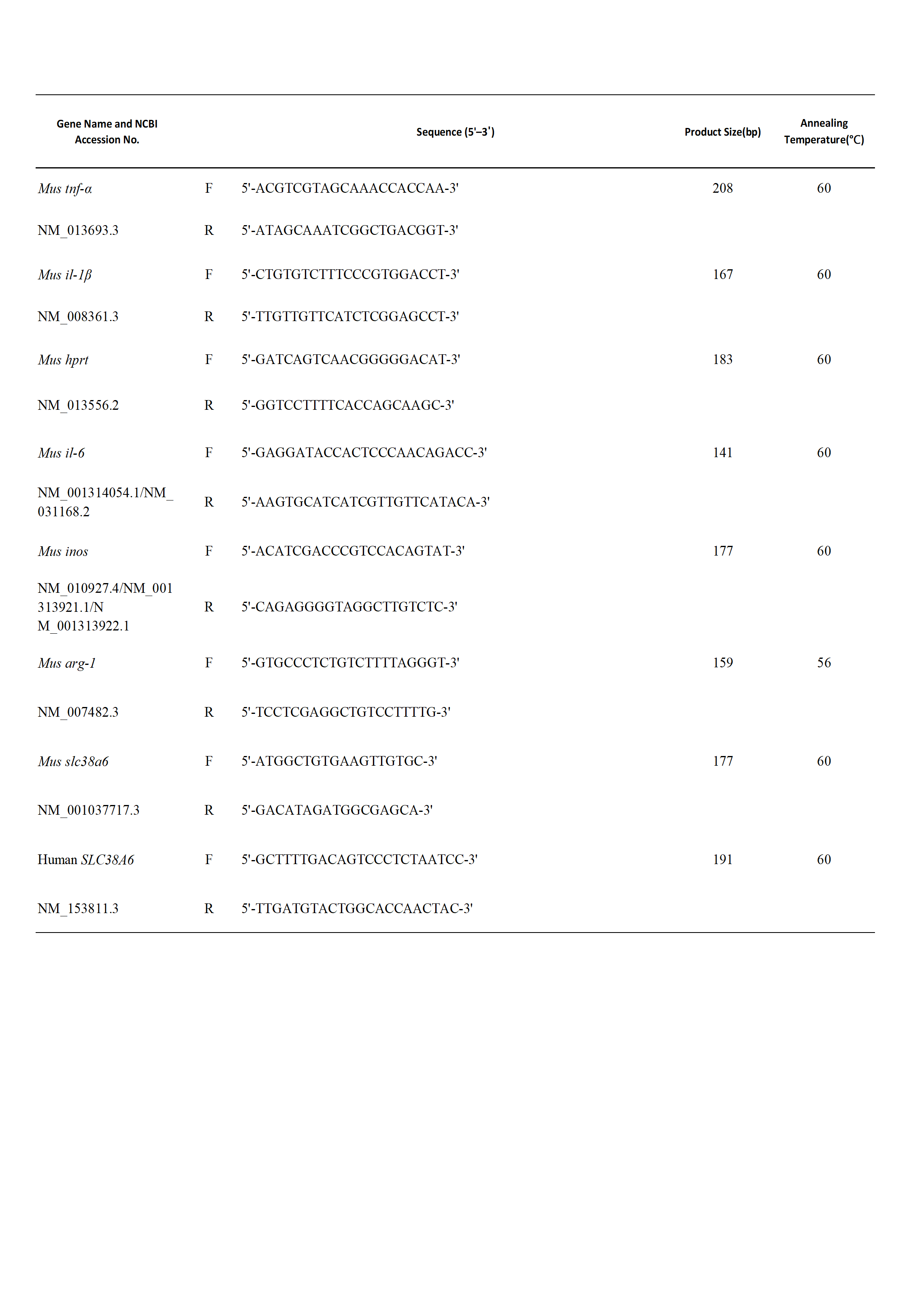


Table S3

The information of mice genotyping primers, products, and hSlc38a6-v2 primers.


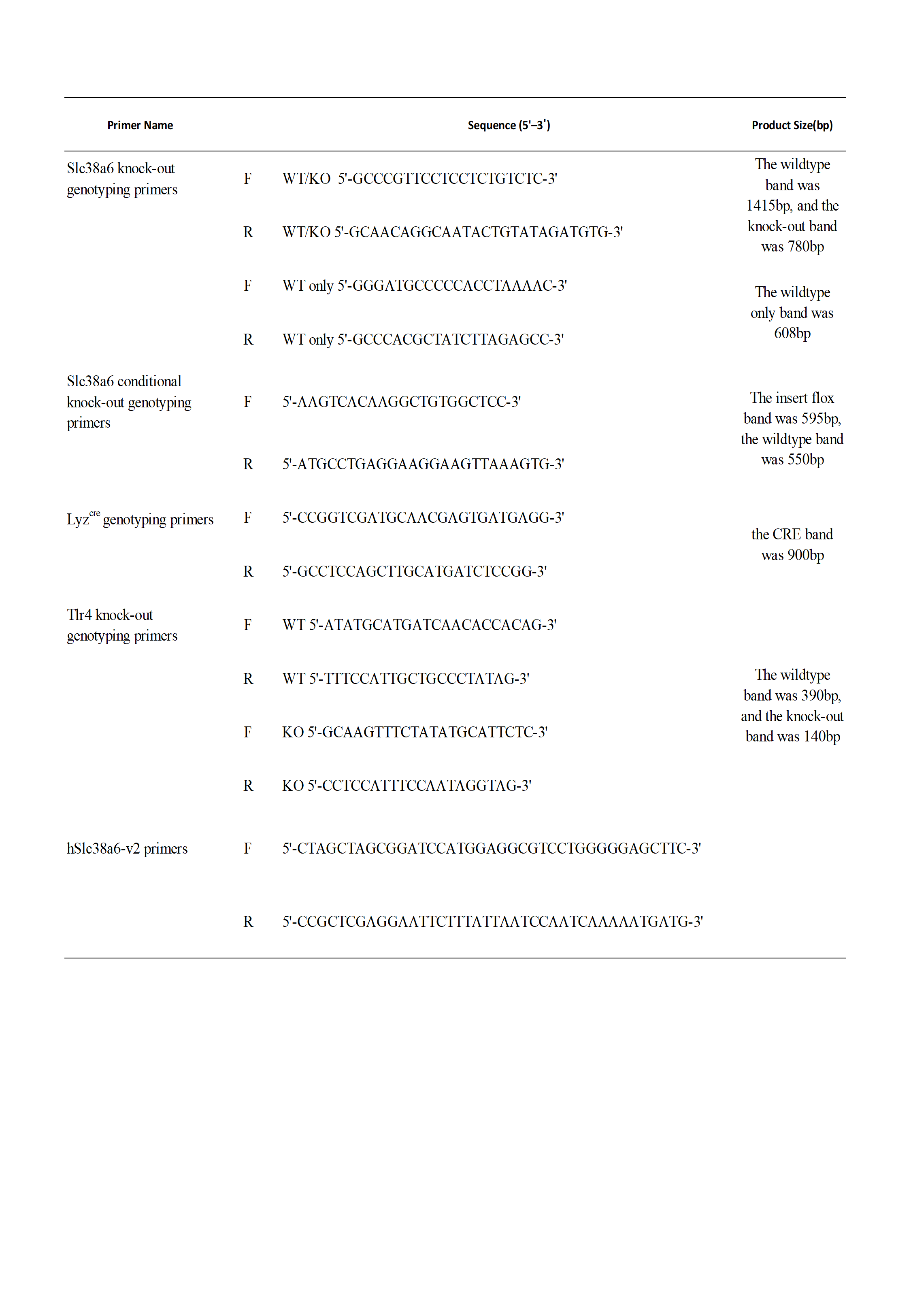


Figure S1


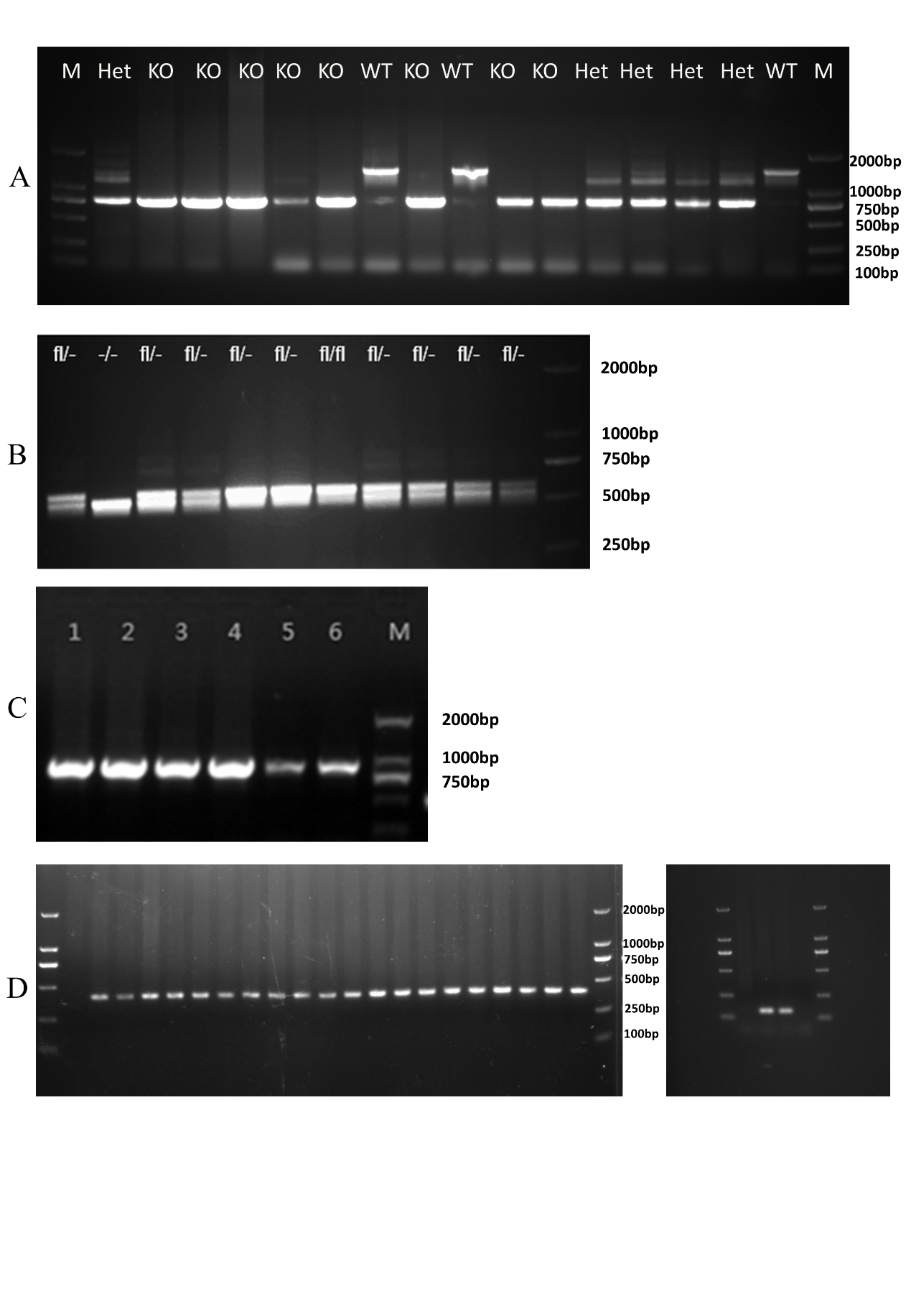
The agarose gel electrophoresis for genotyping. (A) Slc38a6 knock-out mice genotyping. (B) Slc38a6 conditional knock-out mice genotyping. (C) Lyz-CRE mice genotyping. (D) Tlr4 knock-out mice genotyping.

Figure S2


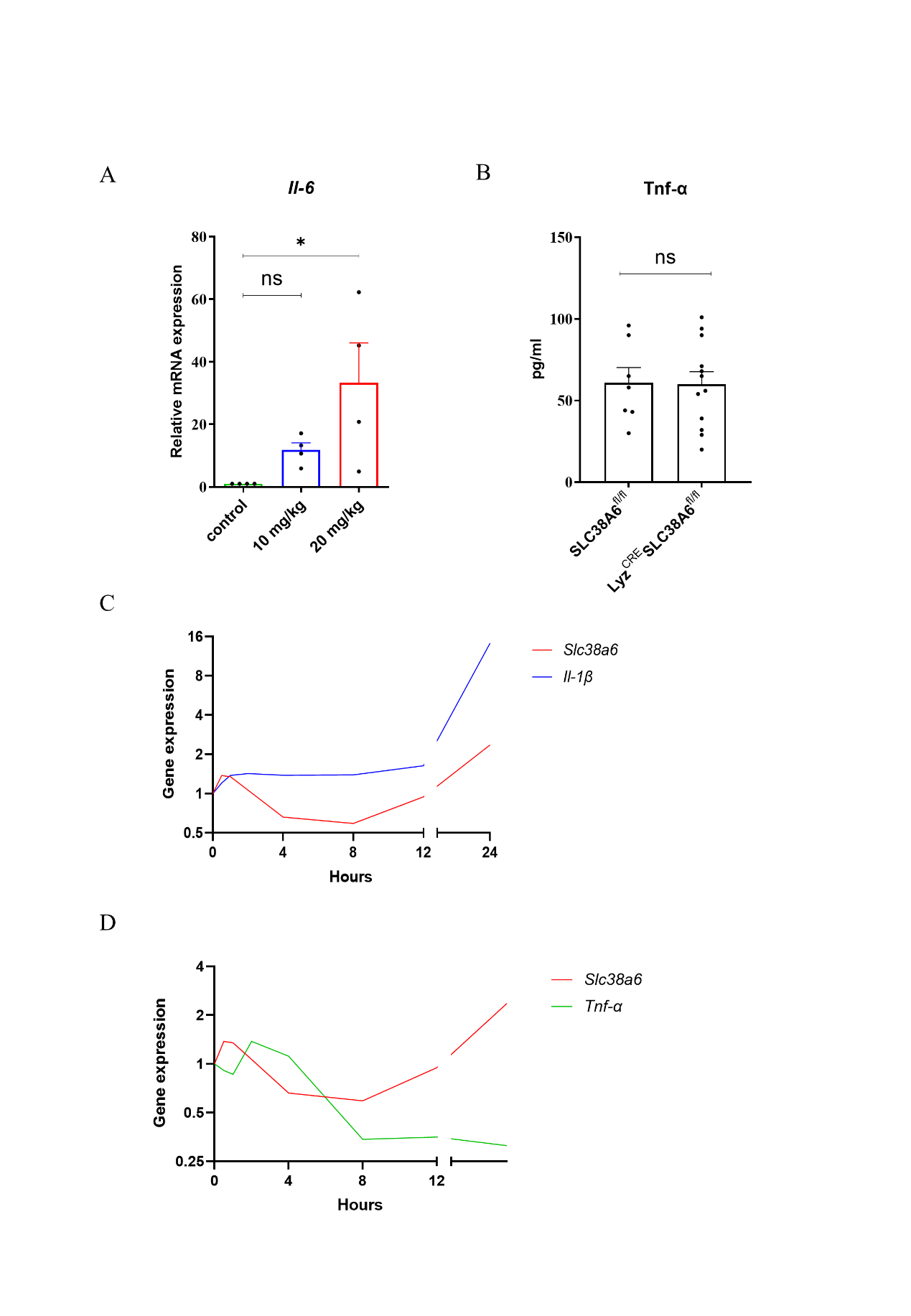
(A) *Il-6* relative expression level in PLF among control group (n=4), 10 mg/kg LPS i.p. group (n= 4) and 20 mg/kg LPS group (n= 4). (B) TNF-α concentration in serum between Lyz^CRE^Slc38a6^fl/fl^ and Slc38a6^fl/fl^ group at 24h post injection by ELISA (n=7 or 12). (C) Relative expression of *Il-1β* and *Slc38a6* after LPS stimulation in RAW264.7. (D) Relative expression of *Tnf-α* and *Slc38a6* after LPS stimulation in RAW264.7. Data are presented as mean ± SEM of independent experiments. *p < 0.05.

Figure S3


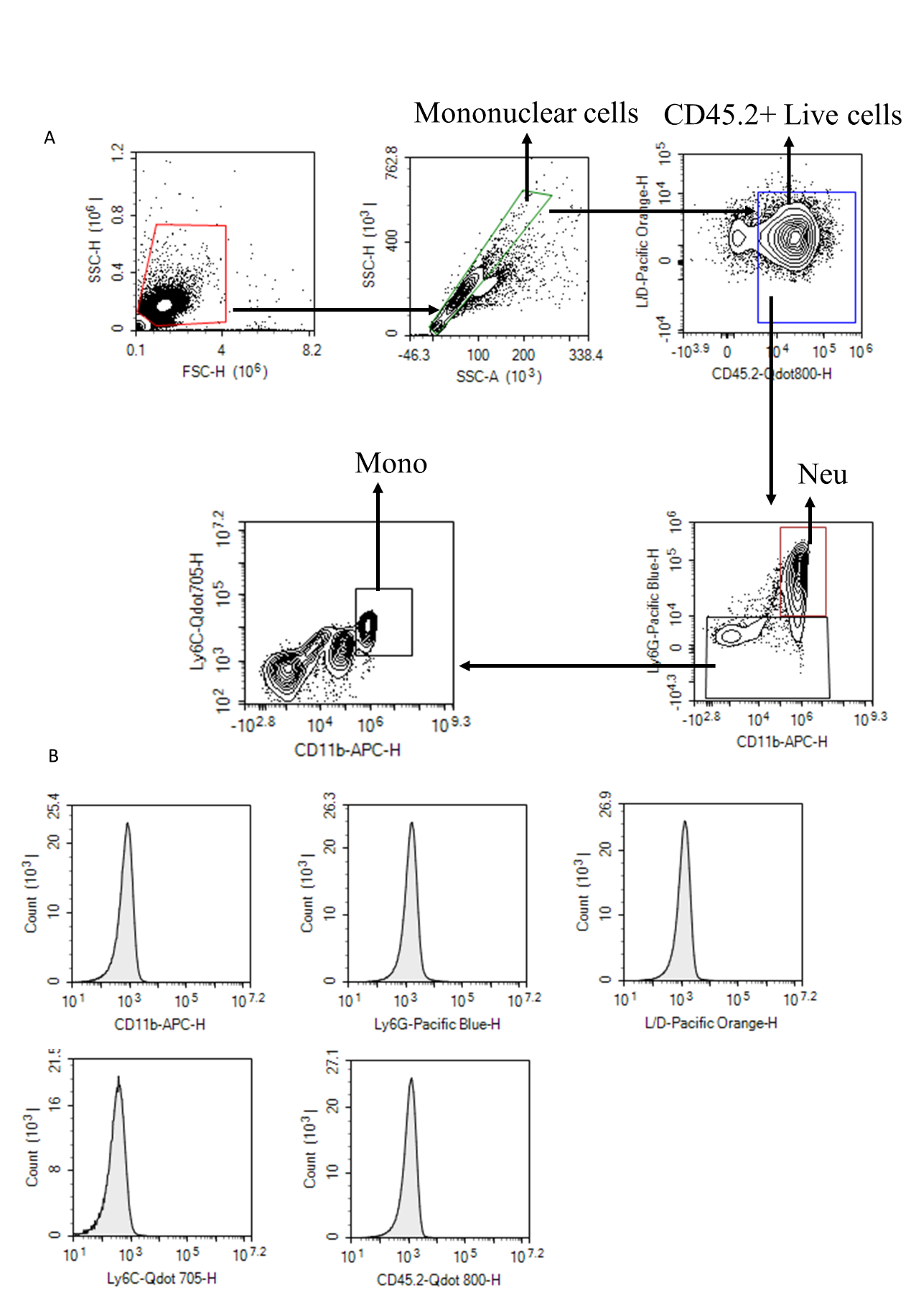
(A) Flow cytometry strategy for peripheral blood. (B) Staining of unstained samples with different antibodies.

Figure S4


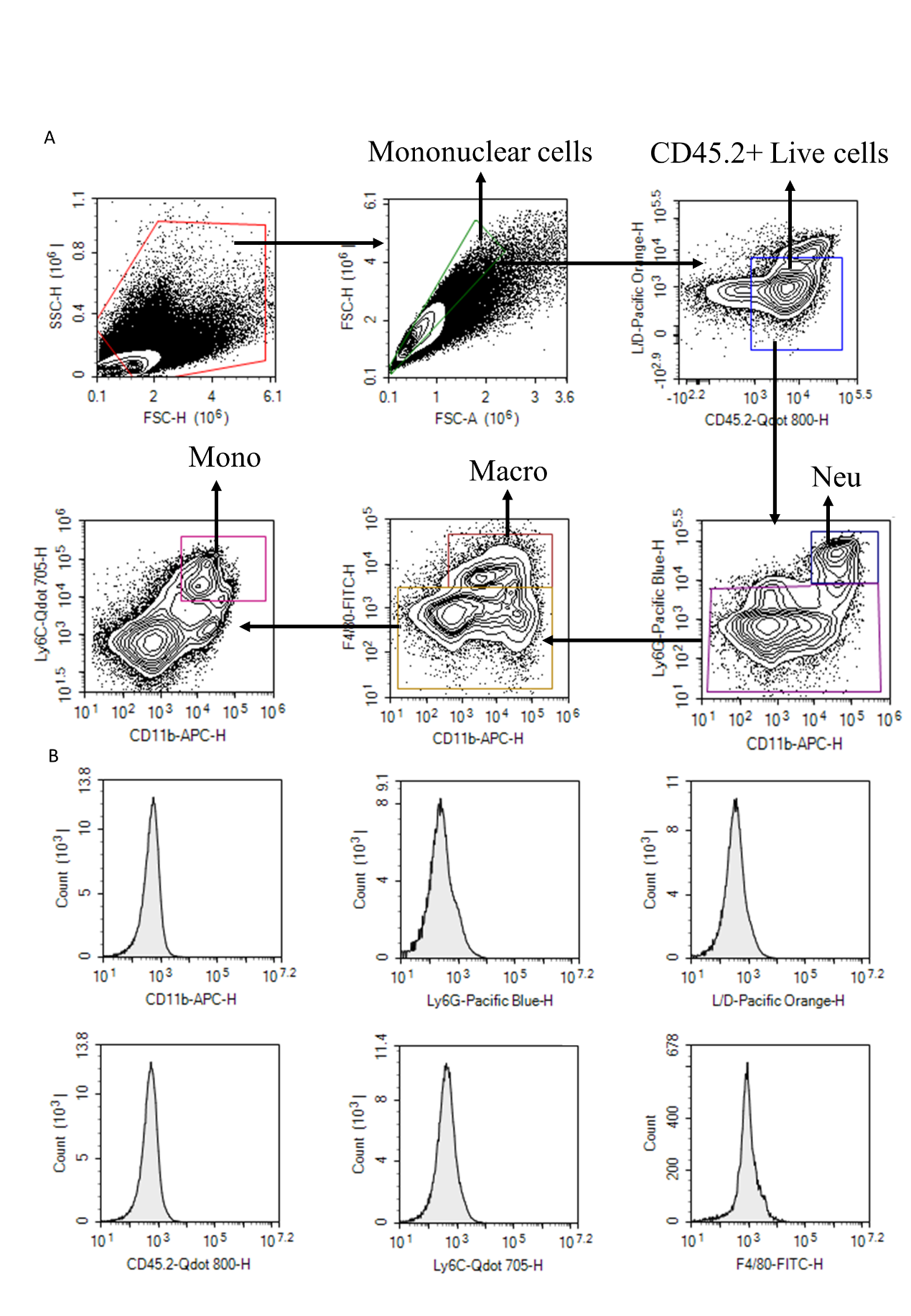
(A) Flow cytometry strategy for spleen cells. (B) Staining of unstained samples with different antibodies.

Figure S5


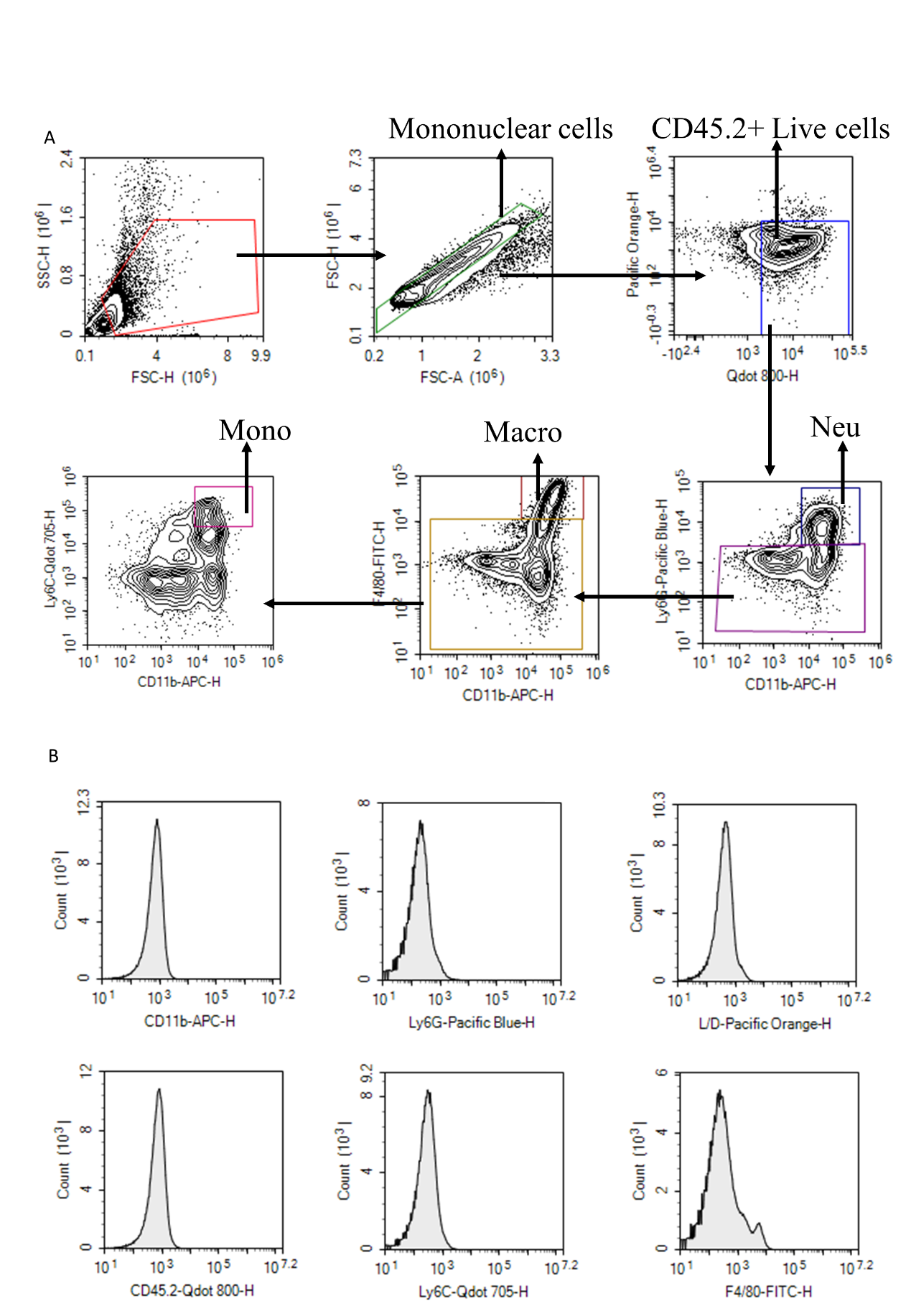
(A) Flow cytometry strategy for PLF. (B) Staining of unstained samples with different antibodies.

Figure S6


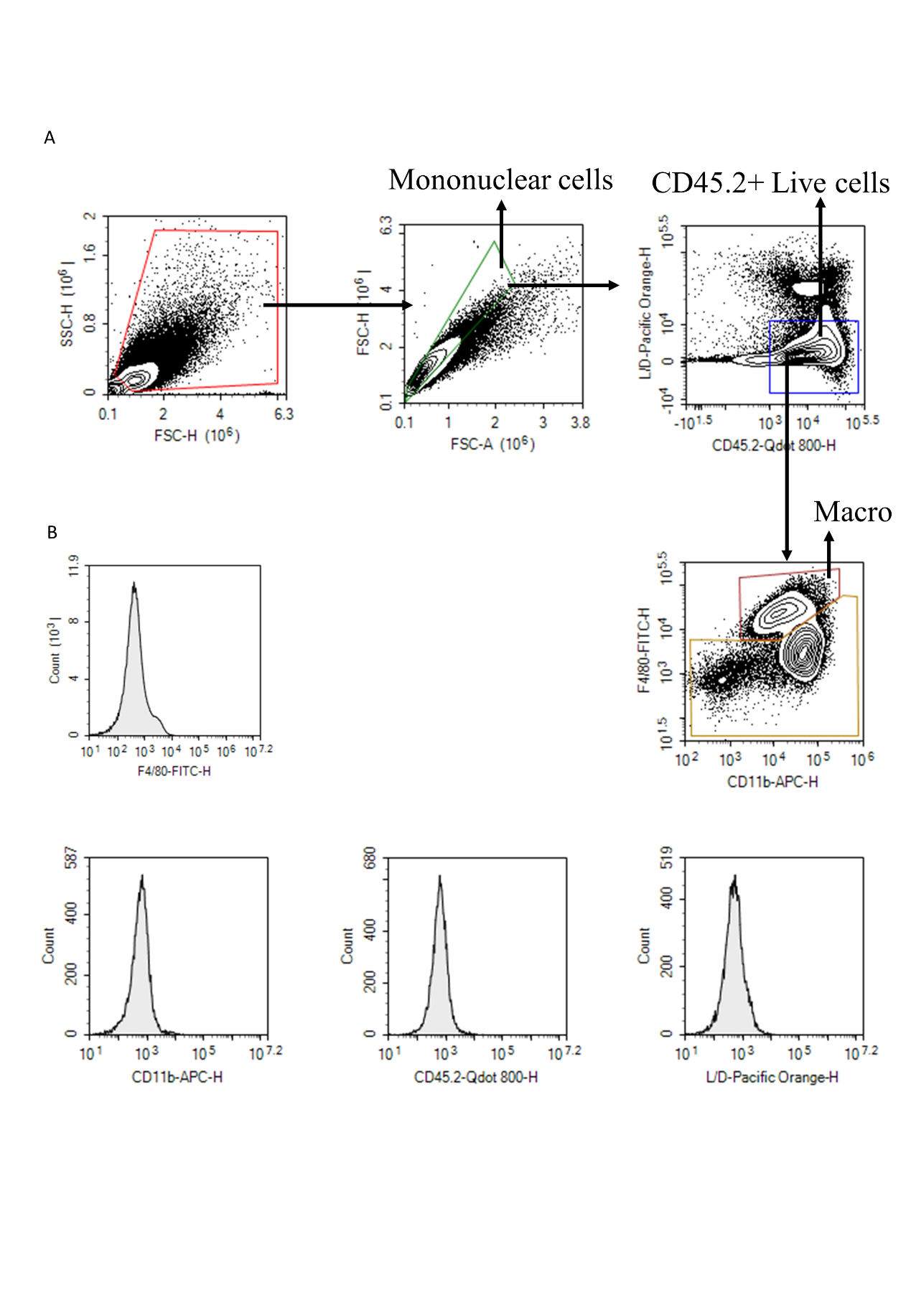
(A) Flow cytometry strategy for BAL fluid. (B) Staining of unstained samples with different antibodies.

Figure S7

Macrophage percentage in BALF between Slc38a6^+/-^ and Slc38a6^-/-^ mice (A) and splenocytes (B) at 72h post injection (n=5). Monocyte/macrophage percentage in splenocytes (C) and in BALF (D) at 72h post injection (n=4-6 or 8).


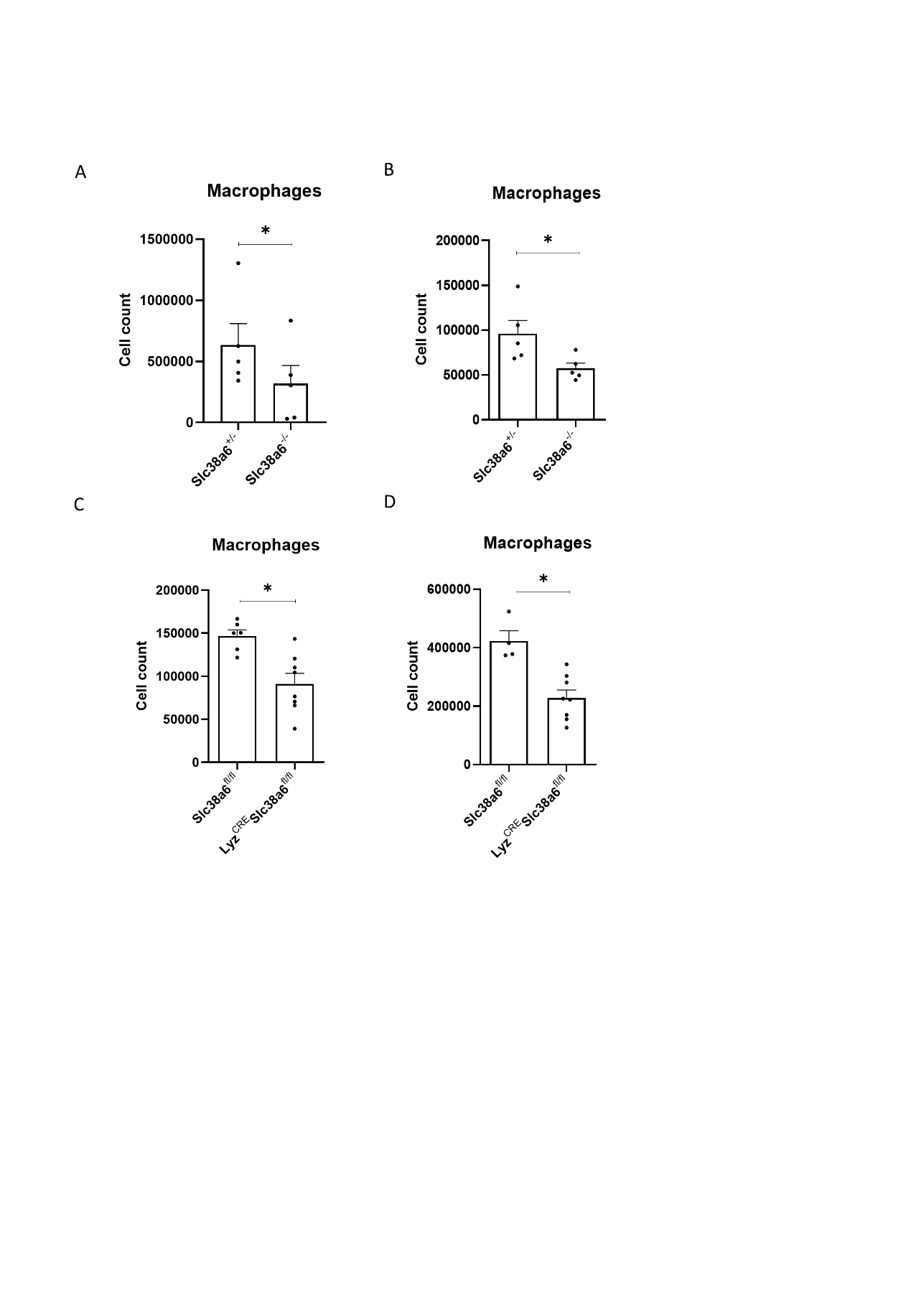

Supplement: Supplementary file 1 — Additional file 1: Table S1. Clinical characteristics of patients. Table S2. The information of primers, products, and annealing temperatures. Table S3. The information of mice genotyping primers, products, and hSlc38a6-v2 primers. Figure S1. The agarose gel electrophoresis for genotyping. (A) Slc38a6 knock-out mice genotyping. (B) Slc38a6 conditional knock-out mice genotyping. (C) Lyz-CRE mice genotyping. (D) Tlr4 knock-out mice genotyping. Figure S2. (A) Il-6 relative expression level in PLF among control group (n = 4), 10 mg/kg LPS i.p. group (n = 4) and 20 mg/kg LPS group (n = 4). (B) TNF-α concentration in serum between LyzCRESlc38a6fl/fl and Slc38a6fl/fl group at 24 h post injection by ELISA (n = 7 or 12). (C) Relative expression of Il-1β and Slc38a6 after LPS stimulation in RAW264.7. (D) Relative expression of Tnf-α and Slc38a6 after LPS stimulation in RAW264.7. Data are presented as mean ± SEM of independent experiments. *p < 0.05. Figure S3. (A) Flow cytometry strategy for peripheral blood. (B) Staining of unstained samples with different antibodies. Figure S4. (A) Flow cytometry strategy for spleen cells. (B) Staining of unstained samples with different antibodies. Figure S5. (A) Flow cytometry strategy for PLF. (B) Staining of unstained samples with different antibodies. Figure S6. (A) Flow cytometry strategy for BAL fluid. (B) Staining of unstained samples with different antibodies. Figure S7. Macrophage percentage in BALF between Slc38a6+/− and Slc38a6−/− mice (A) and splenocytes (B) at 72 h post injection (n = 5). Monocyte/macrophage percentage in splenocytes (C) and in BALF (D) at 72 h post injection (n = 4–6 or 8). [file 12931_2023_2330_MOESM1_ESM.docx]
